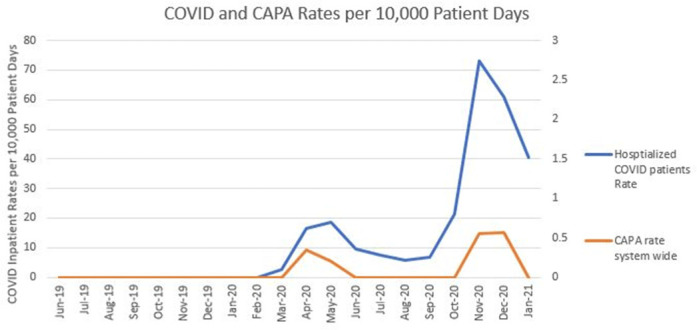# A Cluster of Aspergillosis Associated with SARS-CoV-2

**DOI:** 10.1017/ash.2021.18

**Published:** 2021-07-29

**Authors:** Kerrie VerLee, Jim Codman, Russell Lampen, Chau Nguyen, Tunisia Peters, Greg Kruse, Derek VanderHorst, Doreen Marcinek, Molly Kane-Carbone

## Abstract

**Background:** Coronavirus disease 2019 (COVID-19) has demonstrated a variety of presentations and clinical complications, among them coinfection of pneumonia with the mold *Aspergillus* spp. Patients at risk for invasive disease include transplant recipients and those with prolonged neutropenia, immune disorders, cystic fibrosis, and steroid use. There have been recent descriptions of coronavirus disease–associated pulmonary aspergillosis (CAPA). An outbreak investigation into a cluster of *Aspergillus fumigatus* infections in a health system intensive care unit uncovered a community-onset (CO) increase in CAPA. **Methods:** A multidisciplinary outbreak investigation was conducted evaluating sources of contamination, completion of construction projects, and changes in clinical processes. Retrospective chart review was done for the prior 18 months and incidence density rates for *Aspergillus* infections from June 2019 through December 2020 were calculated per 10,000 patient days, stratified by unit, specimen source, and coinfection with COVID-19. Data were linked with all positive and negative COVID-19 tests performed by the health system’s regional laboratory from March to December 2020. Healthcare-onset (HO) classification was based on infections identified ≥7 days after admission. Statistical analysis was calculated with significance at p < 0.05. **Results:** Over the last 18 months, 82 patients were identified positive with *Aspergillus* cultures; of which 10 (12%) met CAPA definitions. *Aspergillus fumigatus* was the most common species and accounted for 62% of samples, followed by *Aspergillus niger* (17%). Median rates of HO *Aspergillosis* were 0.45 cases per 10,000 patient days, whereas the median total rates of infection were 1.97 cases per 10,000 patient days. Rates of CAPA coincided with COVID-19 hospitalization rates. In the spring and fall, surges of COVID-19, the rate ratio of CAPA to COVID hospitalized infections per 10,000 patient days, ranged from 0.006 to 0.015. Once CAPA infections were adjusted for, rates of CO *Aspergillus* remained high, whereas HO cases suggested baseline acquisition. **Conclusion:** This study outlines rates of CO aspergillosis as well as CAPA rates coinciding with the healthcare system’s spring and fall surges of COVID-19 hospitalizations. Despite the determination that this was not a hospital-acquired cluster, the investigation revealed some areas for opportunity in construction processes along with maintaining coverage of all patient supplies to reduce the risk of contamination.

**Funding:** No

**Disclosures:** None

**Figure 1.**